# Down Syndrome Children, Malocclusion Characteristics and the Need for Orthodontic Treatment Needs (IOTN): A Cross-Sectional Study

**DOI:** 10.3390/children8100888

**Published:** 2021-10-05

**Authors:** Huda Alkawari

**Affiliations:** Department of Pediatric Dentistry and Orthodontics, College of Dentistry, King Saud University, Riyadh 4545, Saudi Arabia; hkawari@ksu.edu.sa; Tel.: +966-505-829-229

**Keywords:** children with Down syndrome DS, index of orthodontic treatment need, malocclusion

## Abstract

The purpose of the present study was to assess the characteristics of malocclusion and determine the orthodontic treatment needs of a group of children with Down syndrome. The study group comprised 23 children aged 10–14 years with Down syndrome who were attending special schools. A clinical examination was performed to measure several parameters that assessed malocclusion as well as classifications based on the Index of Orthodontic Treatment Need (IOTN-DC). When the dental health component (DHC) of the IOTN-DC was considered, results showed that a high percentage of children involved in the current study needed orthodontic treatment (81.9%). Moreover, 59.1% showed Angle’s class-III malocclusion compared to 36.4% who showed class I. However, the differences between the IOTN-DC values for the boys and girls were not statistically significant (*p* > 0.05). The present study has concluded that a higher percentage of children, suffering from Down syndrome, had very severe malocclusion; therefore, treatment can be considered mandatory. Similarly, more than three-fourths of the children with Down syndrome had visited a dental clinic at least once during their life. However, 30.4% of the children’s mothers have mentioned that they had not visited any orthodontic clinic. Therefore, there is a need to develop awareness and knowledge among the parents of children suffering from Down syndrome.

## 1. Introduction

Individuals who need special care and attention are known as special patients [1]. Most such patients include adults and children who tend to have medical issues that often make it difficult to comfortably fit into a regular dental practice. Mostly, they struggle with cooperating or communicating, or suffer from physical restrictions of some form. The majority of the patients in this group include children. There are a variety of special needs conditions and syndromes that may necessitate extra attention from the dentist or their team. In addition to health problems, people with Down syndrome normally experience dental issues, for example, delayed tooth eruption [2]. Furthermore, a higher prevalence of deformed or missing teeth is also common. A routine dental examination is typically difficult for the parent and their carer, as well as the dental team.

Down syndrome (DS or DNS), also referred to as trisomy 21, is a genetic disorder caused by the presence of all or part of a third copy of chromosome 21 [3]. This condition is mostly related to characteristic facial features, physical growth delays, and mild to moderate intellectual disability. Most individuals suffering from Down syndrome are found to suffer from some degree of learning disability. These individuals are at a higher risk of developing other medical conditions. This condition is detected before birth (prenatally) or after birth (postnatally). It cannot be cured but appropriate treatment and care can support individuals with Down syndrome in leading an active life. Several support groups and recommendations are available for Down syndrome patients, their carers, and their families. In Saudi Arabia, the prevalence of Down syndrome has been reported to be 18 per 10,000 live births [4].

Down syndrome patients are also at an increased risk of developing other conditions and medical problems. These include problems with, vision such as abnormal alignment of the eyes (squint); long-sightedness and short-sightedness; and Hearing problems or ear infections. Notably, hearing problems must be detected and treated, as they can affect a child’s ability to learn; as well as impacting bone development, teeth, and growth. Malocclusion is not a disease but a developmental condition on a continuum that represents biological diversity [5]. The treatment of malocclusion is associated with a higher degree of subjectivity and distorted insights about the treatment need [6]. Some of the major traditional reasons for justifying the need for orthodontic treatment are to attain improvement in oral or dental health, in the functioning of the dentition, or in dental or facial aesthetics.

The Index of Orthodontic Treatment Need (IOTN-DC) is often applied to determine those cases that most require orthodontic treatment so that the limited resources can be targeted at individuals with the greatest need and the provision of scarce and expensive treatment for mild cases can be avoided [7]. The objective examination of malocclusion is essential for the documentation of the severity and prevalence of this concern. Most of the indices have been developed to assess malocclusion in a specific community or population [8,9]. The Occlusal Index, based on Angle’s classification is one of the diagnostic indices. This primarily examines the malocclusion incidence in a specific population [9].

In daily clinical practice, however, most decisions concerning treatment originated from the orthodontic treatment need indices. One main reason for orthodontic treatment is a significant improvement in the dental and facial aesthetic. The significance of orthodontic treatment is tough to justify if treatment is founded on improvement in dental or oral health for the majority of orthodontic patients [10]. Studies published in the past demonstrated an amplified awareness of the psychosocial advantage of orthodontic treatment [11]. Primarily, the treatment success is built on the orthodontic indices either after or before treatment. For this reason, the majority of the case presentations rely on Angle’s Occlusal Index. Thus, it leads to misconceptions concerning the use of the indices mentioned above in clinical practice.

A limited number of studies were found in the literature that were aimed at assessing malocclusion characteristics and the need for treatment among children with Down syndrome in Saudi Arabia [12]. The current study findings will, therefore, help better understand the current state of such problems and better estimate future treatment needs. This cross-sectional study aims to assess malocclusion characteristics and orthodontic treatment needs for a group of children with Down syndrome in Riyadh city of Saudi Arabia.

## 2. Materials and Methods

### 2.1. Study Design

A clinical examination was performed to measure different parameters used to assess malocclusion, and the Index of Orthodontic Treatment Need (IOTN-DC) was used to estimate the orthodontic treatment need in this group between June 2020 and May 2021. There are three specialized Down syndrome centers for children in Riyadh city of Saudi Arabia; the study was performed with the Down syndrome Charity Association (DSCA). A total of 23 children with Down syndrome attending the DSCA center were assessed in the study. Additionally, a self-administered questionnaire was provided to the DSCA center’s administration to be distributed to children’s parents and guardians for the purposes of this study.

### 2.2. Target Population/Sample Size

When the study was performed, there were 23 children with Down syndrome within the study target age of 10–14 years old who were attending the DSCA special care center.

### 2.3. Inclusion and Exclusion Criteria

The implemented inclusion criteria were children with Down syndrome attending the DSCA daycare center in Riyadh, and children whose informed consent was obtained from their legal representatives. The exclusion criteria included children not suffering from Down syndrome, children with Down syndrome who were outside of Riyadh city, children with detrimental systemic diseases, children with compound disability, and uncooperative children.

### 2.4. Data Collection/Data Source/Variables

The data for this research study were collected using a self-administered questionnaire followed by a clinical examination. Aside from the demographic profile (age, gender, etc.), medical history, subjective general and oral hygiene practices, and Angle’s classification, several parameters for the assessment of malocclusion and the IOTN-DC were recorded. The examination was undertaken by two calibrated examiners/consultants, who had previously been trained and calibrated in using the IOTN-DC and were working in the field of orthodontics, and inter-examiner reliability was tested (k = 0.86). A disposable examination kit was used, including a mirror, a prop, tweezers, and a cotton roll (all of which were sterile and disposable).

### 2.5. Data Collection/Data Source

A self-administered, structured, validated questionnaire, which included demographic and subjective questions, was completed by the parents/mother or guardian of the participating child. The survey included a few demographic questions, questions on the subjective evaluation of the children’s general and oral health status, Angle’s classification, and the IOTN-DC. The investigator of the study recorded the responses. The Dental Health Component (DHC) of the IOTN-DC has five categories: Grade 1 and Grade 2 represent “no/little need” for orthodontic treatment, Grade 3 represents a “moderate need for treatment”, and Grade 4 and Grade 5 represent a “great/very great need for treatment”.

### 2.6. Data Analysis

The data were entered into a MS Excel spreadsheet. Responses were coded to be analyzed by the Statistical Package for the Social Sciences (SPSS Inc., Chicago, IL, USA), version 17. Qualitative data were presented as frequencies and percentages. 

### 2.7. Ethical Approval

Ethical approval for conducting the study was obtained from the Research and Ethics Committee of the King Khalid University Hospital (date of approval, 29 August 2019 and Approval of Research Project No. E-19-3657, Ref. No. 19/0731/IRB). Consent for contributing to this research was attained from the parents/legal representatives of the children. 

## 3. Results

### 3.1. Sample Overview

**Figure 1:**
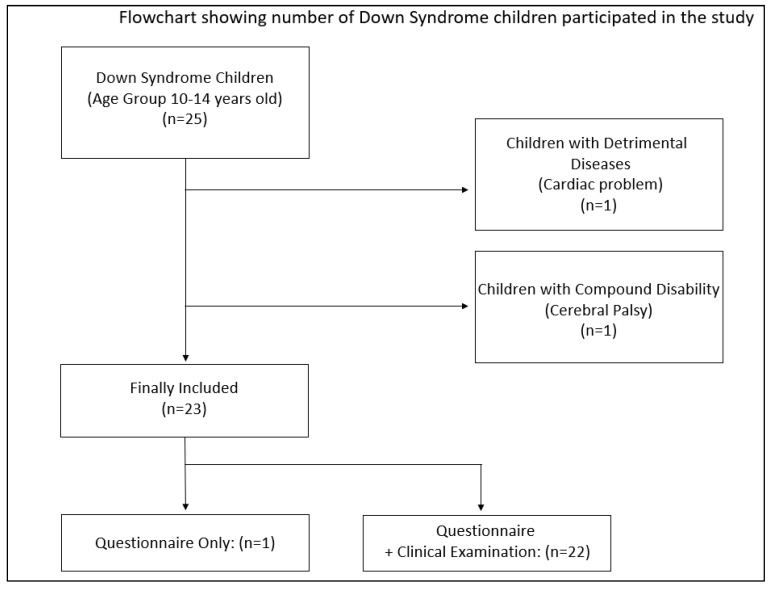
Flowchart showing the number of Down syndrome children participating in the study. (+) Questionnaire and Clinical Examination.

**Figure 2:**
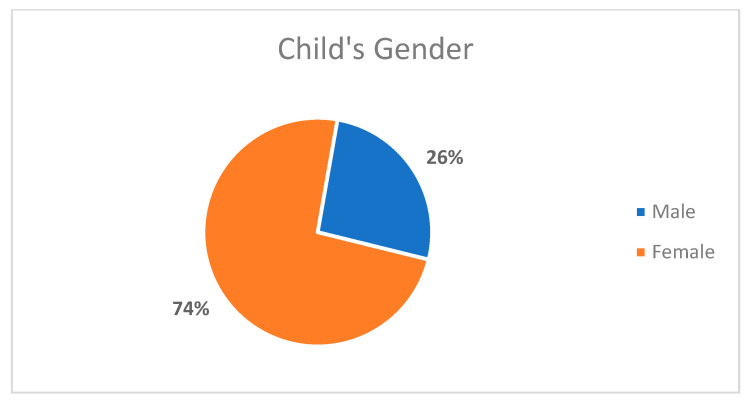
Distribution of the sample of children by gender (*n* = 23).

**Figure 3:**
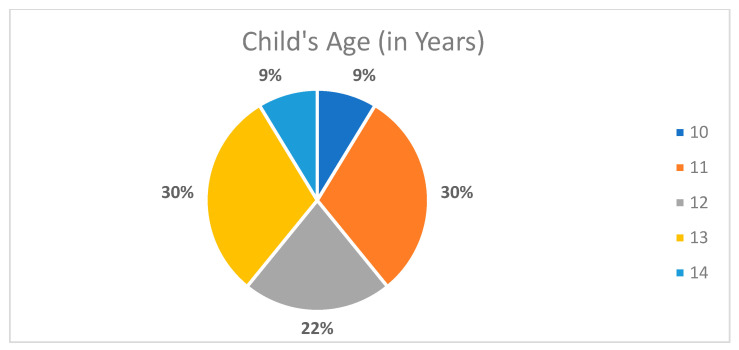
Distribution of the sample of children by age (*n* = 23).

The present research study recruited 25 mothers who provided data about their children. The age group incorporated within the study was children between 10–14 years old. Two children were excluded—one for the presence of detrimental systemic diseases (i.e., a cardiac problem) and another for the presence of compound disability (i.e., cerebral palsy), due to clinical examination difficulty, as specified in the flowchart in Figure 1. Thus, the resulting sample size was 23 children. Overall, 74% of the children were female individuals (Figure 2). Furthermore, the age of the children was also identified. An evaluation of ages also determined that the children and their corresponding mothers were found to have wide-ranging ages. The children were in the age group of 10–14 years, of which 86% of the children were aged between 11 to 13 years. Further details are specified in Figure 3. 

### 3.2. Assessment of the Oral Health Status from Mothers’ Perspective

**Figure 4:**
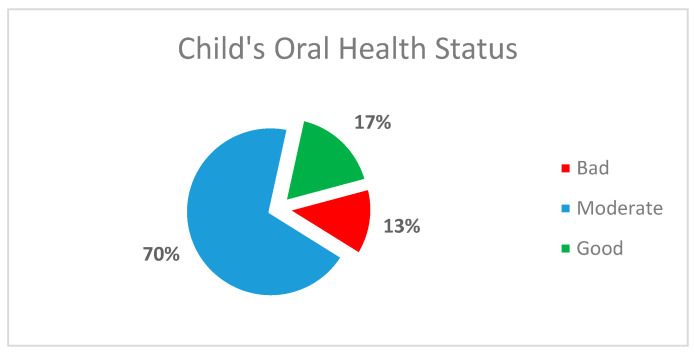
Distribution of the sample of children according to oral health status (*n* = 23).

The present study further identified the mother’s perspectives of the oral health status of their children. A scale of three degrees, including bad, moderate, and good, was used to mark the answers. In response to the assessment of oral health status, 17% of the mothers elucidated their children’s oral health as good. However, only 13% of the mothers mentioned a bad oral health status for their respective children. Further details are mentioned in Figure 4.

### 3.3. Oral Habits

The study further identified the oral habits of the children from their mothers. According to the evaluation, 26% of the children engaged in nail-biting, 22% of the children engaged in finger-sucking, 17% of the children bite their fingers, and 13% of the children engaged in tongue thrust. Meanwhile, only 22% of the children did not engage in any of the listed oral habits. Table 1 displays further details on the oral health habits of the children.

### 3.4. Malocclusion Characteristics

Orthodontic assessment results were also presented in the study. Approximately 59.1% of the children were found to have a class III Angle’s classification, whereas 36.4% showed a class I Angle’s classification. Regarding the overjet, 69.6% of the children were not having increased overjet; however, 30.4% of the children showed an increase in overjet. In terms of the edge-to-edge orthodontic assessment findings, only two children had an edge-to-edge anterior relationship, while 47.9% of the youngsters had reverse anterior overjet. Posterior cross-bite was present in 69.6% of the youngsters; however, 13.1% of the children had scissor bites. When crowding was evaluated, 82.6% of the children had severe crowding, whereas 30.4% of the children had partially erupted teeth. Furthermore, 65.2% of the children had retained deciduous teeth. Lastly, 17.4% of the children were found to have a deep overbite. The details are mentioned in Table 2.

### 3.5. Orthodontic Examination

**Figure 5:**
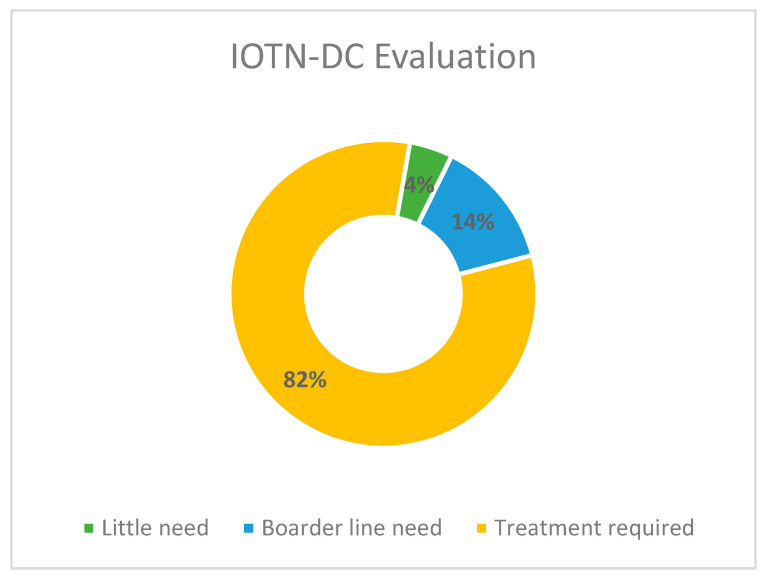
Distribution of the sample of children based on IOTN-DC Evaluation.

An orthodontic examination was also performed in the study, and the results are presented accordingly. A clinical examination was performed among 22 children, after removing the missing data, to measure several parameters for the assessment of malocclusion and the IOTN-DC. Four categories have been developed for the index, which include little need, moderate need, and two categories of groups requiring treatment (great/very great need). The findings indicated that a higher percentage of the children involved in the research study required treatment (81.9%), with 45.5% of the children being categorized within degree 5 of the index. Furthermore, 59.1% of the children had class III malocclusion as compared to 36.4% of children who had class I malocclusion (Table 3 and Figure 5). 

## 4. Discussion

The relevance of using the IOTN-DC index for this study was to assess the need for orthodontic treatment in children with Down syndrome by determining malocclusion features. Some of the limitations of using this index may include a discrepancy between the “Dental Health Component and Aesthetic Component grades” of the IOTN index. The Aesthetic Component of the IOTN evaluates the aesthetic aspects of malocclusion only in the frontal view and only highlights the subjective nature of it.

The findings of the study have identified a higher percentage of children involved in the current study who needed orthodontic treatment (81.9%). Of those who needed treatment, 45.5% of the children had a very high need for treatment, whereas 36.4% of the children required treatment. Furthermore, 13.6% of the children had a moderate need for treatment, and 4.5% of the children had little need for treatment. Moreover, 59.1% showed Angle’s class III malocclusion as compared to 36.4% of children who were marked in class I. However, the differences between the IOTN-DC values for the boys and girls were not statistically significant (*p* > 0.05). 

Such findings have been widely supported by previous research studies. Batista et al. conducted a study on orthodontic treatment in children and adolescents [13]. The study mentioned that prominent upper front teeth are a common problem that affects the majority of the children in the United Kingdom. Therefore, orthodontic treatment has been assessed among children. The findings of the study have supported the outcomes of the current study and have mentioned that most children need orthodontic treatment. Moreover, work completed by Doriguetto et al. has also supported the outcomes of the current study [14]. The present study aimed to identify malocclusion among children and adolescents suffering from Down syndrome. Previous studies have shown a higher prevalence rate of malocclusion among patients with Down syndrome as compared to individuals not having Down syndrome. Therefore, this study aimed to evaluate whether children with Down syndrome are more affected by malocclusion or not. According to Luconi et al., there is no correlation between malocclusion and bruxism, which proves that there is no biological significance between the conditions, and hence, malocclusion does not increase the probability of bruxism among children with Down syndrome [15]. Based on the previously published systematic review, findings indicated that malocclusion was more prevalent among children suffering from Down syndrome; therefore, orthodontic treatment needs are also higher among children suffering from Down syndrome. The current study is based on primary data; therefore, the study conducted by Doriguetto et al. appropriately verifies the outcomes of the current study [14]. 

A recent study conducted by Dewi et al. aimed to identify dental and oral care treatment needs among children suffering from Down syndrome. This study also collected secondary data regarding the dental and oral examination of 34 children with Down syndrome who were aged 5 to 17 years. The results of the study suggested that most of the children had orthodontic treatment needs [16]. Moreover, most of the needs were related to restorations and extractions. This study has also supported the outcomes of the current study; however, the perspective related to restoration and extractions has not been identified in the current study. Further, the relevance of oral problems in children with Down syndrome was explored by Pini DM et al., indicating that malocclusion class I, in addition to inadequate oral hygiene, was observed in children with Down syndrome [17]. Aghimien et al. also identified the prevalence of malocclusion among individuals suffering from Down syndrome. This study has mainly covered all individuals suffering from Down syndrome; however, the present study only considered children. According to the findings of this study, class III skeletal patterns was highly significant among individuals suffering from Down syndrome. Moreover, the study also concluded that the need for orthodontic treatment is higher among individuals suffering from Down syndrome [18]. Therefore, it can be said that this recent study has also supported the outcomes of the current study.

Another study has been conducted by Alkhabuli et al., which aimed to identify the oral health status and treatment needs of children with special needs. The findings of the study primarily identified that there was a higher prevalence of dental caries and periodontal diseases among the recruited patients. The study highly recommended the education of children’s parents and caregivers on the need for a proper diet, proper oral hygiene, and dental visits [19]. Therefore, the outcomes of this study can be said to be relevant to the current study. Meanwhile, Alkhadra et al. conducted a comprehensive study in Saudi Arabia to identify the characteristics of malocclusion among children with special needs. The findings of the study are an exact match with the outcomes of the current study [12]. It has been identified that the children suffering from Down syndrome had a higher incidence rate of class III malocclusion. The findings of the current study showed that 59.1% of the children have shown Angle’s class III malocclusion. Therefore, the perspective of malocclusion has also been supported by previous data. In addition, this study collected primary data from the city of Riyadh, Saudi Arabia. Therefore, the outcomes of this study are similar to that of the current study [12]. In other words, the current study is examining the relevance of malocclusion in children with Down syndrome, which is affecting their quality of life and should be touched upon thoroughly. Vesna et al. conducted a comprehensive study, which aimed to identify the dental aspects among children suffering from Down syndrome. This study is essentially a case report wherein dental aspects have been assessed in patients with Down syndrome. The study’s findings mentioned that malocclusion was extremely common among patients; therefore, the needs for orthodontic treatment were also higher among the patients [20]. Therefore, it can be said that the findings of this study can be correlated with the outcomes of the current study. For future studies on this subject of orthodontic treatment for children with Down syndrome, a larger sample size is recommended. As this study was performed during the COVID-19 pandemic, the collection of more samples for clinical data was not possible due to the closures of Down syndrome centers in order to preserve the children’s health.

## 5. Conclusions

The assessment of malocclusion and treatment needs (IOTN-DC) concludes that a higher percentage of children suffering from Down syndrome had severe malocclusion; therefore, treatment can be considered mandatory. Hence, there is a need to develop awareness among the parents and guardians of children with Down syndrome. Awareness should be provided in regards to oral examination and regular visits to orthodontic clinics for required orthodontic treatment. These elements will go a long way in helping children reduce the severity of malocclusion and improving their physical needs and aesthetic features. With such a large demand for special orthodontic treatment for children with Down syndrome, it is essential to establish a centralized service-providing entity to cater to such an important need, so that treatment for children with such distinct cases is prioritized.

## Figures and Tables

**Table 1:** Descriptive Statistics of Children’s Oral Habits (*n* = 23).

Variable	Number *(%)*
Child’s Oral Habits	
Nail-biting	6 (26.0)
Finger sucking	5 (22.0)
Finger biting	4 (17.0)
Tongue thrust	3 (13.0)
None	5 (22.0)

**Table 2:** Orthodontic Assessment Results.

Variable	Number (%)
**Angle’s Classification (*n* = 22)**	
I	8 (36.4)
II	1 (0.6)
III	13 (59.1)
**Overjet (*n* = 23)**	
Yes	7 (30.4)
None	16 (69.6)
**Anterior Relationship (Edge to edge) (*n* = 23)**	
Yes	2 (8.7)
None	21 (91.3)
**Reverse of Overjet (*n* = 23)**	
Yes	11 (47.9)
None	12 (52.1)
**Local Anterior Cross-Bite (*n* = 23)**	
Yes	16 (69.6)
None	7 (30.4)
**Crowding (*n* = 23)**	
Yes	19 (82.6)
None	4 (17.4)
**Posterior Cross-Bite (*n* = 23)**	
Yes	11 (47.8)
None	12 (52.2)
**Scissor-Bite (*n* = 23)**	
Yes	3 (13.1)
None	20 (86.9)
**Partially Erupted Teeth (*n* = 23)**	
Yes	7 (30.4)
None	16 (69.6)
**Retained Deciduous Teeth (*n* = 23)**	
Yes	15 (65.2)
None	8 (34.8)
**Congenitally Missing Teeth (*n* = 23)**	
Yes	3 (13.1)
None	20 (86.9)
**Deep Overbite (*n* = 23)**	
Yes	4 (17.4)
None	19 (82.6)

**Table 3:** Orthodontic Assessment Results.

Variable	Frequency	Percent (%)
IOTN-DC (*n* = 22)		
2—Little need	1	4.5
3—Moderate need	3	13.6
4—Treatment required (great need)	8	36.4
5—Treatment required (very great need)	10	45.5

## References

[1] Davis A.M., Brown R.F., Taylor J.L., Epstein R.A., McPheeters M.L. (2014). Transition care for children with special health care needs. Pediatrics.

[2] Skotko B.G., Levine S.P., Goldstein R. (2011). Self-perceptions from people with Down syndrome. Am. J. Med Genet. Part. A.

[3] Patterson D. (2009). Molecular genetic analysis of Down syndrome. Hum. Genet..

[4] Salloum A.A., El Mouzan M.I., Al Herbish A., AlOmer A., Qurashi M. (2015). Prevalence of selected congenital anomalies in Saudi children: A community-based study. Ann. Saudi Med..

[5] Proffit W.R., Fields H.W., Sarver D.M. (2006). Contemporary Orthodontics.

[6] Richmond S., O’Brien K., Buchanan I., Burden D. (1992). An Introduction to Occlusal Indices.

[7] Borzabadi-Farahani A. (2012). A review of the oral health-related evidence that supports the orthodontic treatment need indices. Prog. Orthod..

[8] So L.L., Tang E.L. (1993). A comparative study using the Occlusal Index and the Index of Orthodontic Treatment Need. Angle Orthod..

[9] Borzabadi-Farahani A. (2011). An insight into four orthodontic treatment need indices. Prog. Orthod..

[10] Burden D.J. (2007). Oral Health-Related Benet s of Orthodontic Treatment. Semin. Orthod..

[11] Hunt O., Hepper P., Johnston C., Stevenson M., Burden D. (2001). Professional perceptions of the benefits of orthodontic treatment. Eur. J. Orthod..

[12] Alkhadra T. (2017). Characteristic of malocclusion among Saudi special need group children. J. Contemp. Dent. Pract..

[13] Batista K.B., Thiruvenkatachari B., Harrison J.E., O’Brien K.D. (2018). Orthodontic treatment for prominent upper front teeth (Class II malocclusion) in children and adolescents. Cochrane Database Syst. Rev..

[14] Doriguetto P.V., Carrada C.F., Scalioni F.A., Abreu L.G., Devito K.L., Paiva S.M., Ribeiro R.A. (2019). Malocclusion in children and adolescents with Down syndrome: A systematic review and meta-analysis. Int. J. Pediatric Dent..

[15] Luconi E., Togni L., Mascitti M., Tesei A., Nori A., Barlattani A., Procaccini M., Santarelli A. (2021). Bruxism in Children and Adolescents with Down Syndrome: A Comprehensive Review. Medicina.

[16] Dewi A.M., Saskianti T., Puteri M.M. (2021). Dental and Oral Care Treatment Needs in Children with Down Syndrome in Surabaya. Indian J. Forensic Med. Toxicol..

[17] Pini D.M., Fröhlich P.C., Rigo L. (2016). Oral health evaluation in special needs individuals. Einstein.

[18] Aghimien O., Ajayi E., Ize-Iyamu I.N. (2021). Prevalence of Malocclusion in Down Syndrome Individuals in Benin City, Nigeria. Niger. J. Med Dent. Educ..

[19] Alkhabuli J.O., Essa E.Z., Al-Zuhair A.M., Jaber A.A. (2020). Oral Health status and treatment needs for children with special needs: A cross-sectional study. Pesqui. Bras. Odontopediatria E Clín. Integr..

[20] Vesna A., Ivkovska A.S., Stavreva N. (2017). Dental aspects of children with Down syndrome. J. Dent. Probl. Solut..

